# Re: “Moving Forward After Two Deaths in a Gene Therapy Trial of Myotubular Myopathy” by Wilson and Flotte

**DOI:** 10.1089/hum.2020.217

**Published:** 2020-08-17

**Authors:** Perry B. Shieh, Carsten G. Bönnemann, Wolfgang Müller-Felber, Astrid Blaschek, James J. Dowling, Nancy L. Kuntz, Andreea M. Seferian

**Affiliations:** ^1^ University of California at Los Angeles School of Medicine, Los Angeles, California, USA; ^2^ National Institute of Neurological Disorders and Stroke, National Institutes of Health, Bethesda, Maryland, USA; ^3^ Klinikum der Universität München, Munich, Germany; ^4^ Hospital for Sick Children, Toronto, Ontario, Canada; ^5^ Ann and Robert H. Lurie Children's Hospital of Chicago, Chicago, Illinois, USA; ^6^ Institute of Myology, Pitié-Salpêtrière Hospital, Paris, France.

**Dear Editor,**

In response to the recent editorial, “Moving Forward After Two Deaths in a Gene Therapy Trial of Myotubular Myopathy,” and as investigators for the ASPIRO study in children with X-linked myotubular myopathy (XLMTM), we want to clarify discrepancies in the information provided and emphasize several points. We do so in the spirit of transparency for the scientific community that continues to pursue gene therapies to safely improve the lives of patients.

ASPIRO is a first-in-human study of intravenously administered AT132, an investigational therapeutic *MTM1* (myotubularin) gene transfer using a recombinant AAV8 vector for enhanced muscle transduction and a muscle-specific promoter for tissue-restricted expression. Preclinical studies of AT132 were conducted by Audentes in two animal models of XLMTM (*MTM1* knockout mouse and a naturally occurring missense-mutated canine) and in wild-type nonhuman primates (NHPs). These studies showed that doses of AT132 up to 8 × 10^14^ vg/kg (vector genomes per kg) were well tolerated in NHPs with no adverse findings related to AT132 exposure (unpublished data).

Doses for the ASPIRO ascending-dose clinical trial were informed by efficacy and safety observations from those carefully designed animal experiments. Importantly, the hepatotoxicity observed in three boys who received the 3 × 10^14^ vg/kg dose in ASPIRO was not observed in the XLMTM animals and in NHPs that received 8 × 10^14^ vg/kg.

To date, 17 boys have received AT132 at 3 × 10^14^ vg/kg, in the initial dose escalation cohort of ASPIRO or in a later expansion cohort, which proceeded after careful assessment of prior dosed patients, including by an independent monitoring committee. Two of the 17 boys who received AT132 at the 3 × 10^14^ vg/kg dose experienced fatal liver dysfunction and one has ongoing severe liver dysfunction. These three boys, who were in the later expansion cohort, shared several notable features: they were older, heavier (and thus received among the highest total vg, range: 4.80 × 10^15^–7.74 × 10^15^ total vg), and all had evidence of likely pre-existing intrahepatic cholestasis. Although the two deaths occurred months after dosing, all three boys demonstrated signs of liver dysfunction within 3–4 weeks after receiving AT132.

Although the pathomechanism is not yet characterized, hepatobiliary disease is observed in the XLMTM population.^1–3^ After treatment with AT132, liver findings in these three boys included intrahepatocellular and canalicular cholestasis, periportal and bile ductular reaction, secondary fibrosis, and notable lack of prominent liver parenchymal inflammatory cellular infiltrates. More than 50% of subjects in the ASPIRO trial have some evidence of pre-existing hepatobiliary disease—including intermittent direct hyperbilirubinemia, intermittent transaminase elevation, and/or historical cholestasis or jaundice—yet no similar, treatment-related, serious cholestatic liver dysfunction has been observed in the six patients in the 1 × 10^14^ vg/kg cohort or in any of the younger, lighter patients in the 3 × 10^14^ vg/kg cohort regardless of pre-existing hepatobiliary disease.

It is important to evaluate whether immune mechanisms may have contributed to the severe liver dysfunction observed, particularly in light of what has been published for adeno-associated virus-mediated gene therapy across varying serotypes and dose levels, including in preclinical settings. However, full investigations of these events are ongoing, and it is premature to draw definitive conclusions at this time.

We have observed favorable efficacy data for gene transfer therapy in murine and canine XLMTM, with neither animal study suggesting adverse findings related to hepatobiliary disease. In 2019, we presented preliminary efficacy and safety data from ASPIRO, highlighting the potential for AT132 to transform the lives of children with this fatal and life-limiting disease.^4^ As we continue to learn more about all of the patients from ASPIRO, our intention is to publish the full data set as soon as is practicable.

These children and their families have indeed shown great bravery. We honor that bravery by exploring the underlying etiology of these events as diligently as possible and remain committed to delivering to them a safe and effective therapy.
